# Efficacy and *In Vitro* Activity of Novel Antibiotics for Infections With Carbapenem-Resistant Gram-Negative Pathogens

**DOI:** 10.3389/fcimb.2022.884365

**Published:** 2022-05-20

**Authors:** Flora Cruz-López, Adrian Martínez-Meléndez, Rayo Morfin-Otero, Eduardo Rodriguez-Noriega, Héctor J. Maldonado-Garza, Elvira Garza-González

**Affiliations:** ^1^Subdirección Académica de Químico Farmacéutico Biólogo, Facultad de Ciencias Químicas, Universidad Autónoma de Nuevo León, San Nicolás de los Garza, Mexico; ^2^Instituto de Patología Infecciosa y Experimental “Dr. Francisco Ruiz Sánchez”, Centro Universitario de Ciencias de la Salud, Universidad de Guadalajara, Guadalajara, Mexico; ^3^Servicio de Gastroenterología, Hospital Universitario 'Dr. José Eleuterio González', Universidad Autónoma de Nuevo León, Monterrey, Mexico; ^4^Laboratorio de Microbiología Molecular, Departamento de Bioquímica y Medicina Molecular, Facultad de Medicina, Universidad Autónoma de Nuevo León, Monterrey, Mexico

**Keywords:** carbapenemase, drug resistance, new antibiotics, acinetobacter, gram negatives

## Abstract

Infections by Gram-negative multi-drug resistant (MDR) bacterial species are difficult to treat using available antibiotics. Overuse of carbapenems has contributed to widespread resistance to these antibiotics; as a result, carbapenem-resistant Enterobacterales (CRE), *A. baumannii* (CRAB), and *P. aeruginosa* (CRPA) have become common causes of healthcare-associated infections. Carbapenems, tigecycline, and colistin are the last resource antibiotics currently used; however, multiple reports of resistance to these antimicrobial agents have been documented worldwide. Recently, new antibiotics have been evaluated against Gram-negatives, including plazomicin (a new aminoglycoside) to treat CRE infection, eravacycline (a novel tetracycline) with *in vitro* activity against CRAB, and cefiderocol (a synthetic conjugate) for the treatment of nosocomial pneumonia by carbapenem-non-susceptible Gram-negative isolates. Furthermore, combinations of known β-lactams with recently developed β-lactam inhibitors, such as ceftazidime-avibactam, ceftolozane-tazobactam, ceftazidime-tazobactam, and meropenem-vaborbactam, has been suggested for the treatment of infections by extended-spectrum β-lactamases, carbapenemases, and AmpC producer bacteria. Nonetheless, they are not active against all carbapenemases, and there are reports of resistance to these combinations in clinical isolates.This review summarizes and discusses the *in vitro* and clinical evidence of the recently approved antibiotics, β-lactam inhibitors, and those in advanced phases of development for treating MDR infections caused by Gram-negative multi-drug resistant (MDR) bacterial species.

## 1 Introduction

Emerging multi-drug resistant organisms are a threat to healthcare systems worldwide, especially as causative agents of hospital-acquired infections (HAIs) (Nekkab et al., 2017; Lee et al., 2021). The most frequently occurring HAIs are urinary tract infection (UTI), intra-abdominal infection (IAI), ventilator-associated pneumonia (VAP), and bloodstream infection (BSI). Drug-resistant Gram-negative bacilli—including Enterobacterales (mainly *Escherichia coli* and *Klebsiella pneumoniae*), *Pseudomonas aeruginosa*, and *Acinetobacter baumannii—*frequently cause these infections, although these species also can colonize the respiratory tract (Smith et al., 2004; Nekkab et al., 2017).

Carbapenems, tigecycline, and colistin are currently used to treat infections caused by some of these microorganisms (Harris et al., 2018; Hayden and Won, 2018). Unfortunately, overuse of carbapenems has contributed to widespread resistance to this antibiotic; as a result, carbapenem-resistant Enterobacterales (CRE), *A. baumannii* (CRAB), and *P. aeruginosa* (CRPA) have become common causes of healthcare-associated infections (Logan and Weinstein, 2017; Aung et al., 2021; Palacios-Baena et al., 2021). Treatment options for infections associated with CRE are limited, and these infections are associated with high clinical failure, morbidity, and mortality, especially among patients in the intensive care unit (ICU) (Logan and Weinstein, 2017; Aung et al., 2021; Palacios-Baena et al., 2021). At present, hospital outbreaks associated with CRE have been reported (Baran and Aksu, 2016; van Loon et al., 2018).

CRPA is a significant public health threat that has few treatment options. The frequency of infection by this bacterial species has been reported to be up to 9.8% (Tamma et al., 2017; Chen et al., 2019; Walters et al., 2019). Difficult-to-treat resistance in *P. aeruginosa* is defined as clinical isolates that are non-susceptible to all of the following treatments: piperacillin-tazobactam, ceftazidime, cefepime, aztreonam, meropenem, imipenem-cilastatin, ciprofloxacin, and levofloxacin (Magiorakos et al., 2012; Kadri et al., 2018). Recently, cefiderocol and BL-BLI combinations such as ceftazidime/avibactam and ceftolozane-tazobactam are recommended to treat CRPA infections (Kadri et al., 2018; Tamma et al., 2021a; Tamma et al., 2021b).

Of all carbapenem-resistant Gram-negative infections, *A. baumannii* accounts for up to 22% (Cai et al., 2017; Perez et al., 2020). *A. baumannii* infection affects patients in ICUs and is frequently associated with VAP and BSI (Peleg et al., 2008). Globally, there are approximately one million annual cases of *A. baumannii* infection; approximately 50% of these infections are caused by *A. baumannii*, which is resistant to multiple antibiotics, including carbapenems (Spellberg and Rex, 2013). Numerous reports of CRAB have been published worldwide, and frequencies of CRAB as high as 90% have been reported (Xie et al., 2018; Isler et al., 2019; Piperaki et al., 2019; Garza-González et al., 2020; López-Jácome et al., 2021). The antimicrobial agents used to treat CRAB infections include polymyxins, tigecycline, and sulbactam. Other antibiotics—including minocycline, trimethoprim-sulfamethoxazole, rifampicin, fosfomycin, and aminoglycosides—have been used with limited efficacy and pharmacokinetics disadvantages (Piperaki et al., 2019). Additionally, researchers have reported CRAB infections that are resistant to all antibiotic classes, excluding polymyxins and tigecycline (extensively drug-resistant, XDR) and CRAB strains that are resistant to colistin and tigecycline (pan-resistant) (Wong et al., 2017).

## 2 Drug Resistance and Genes Encoding Carbapenemases

Drug resistance is generated by the combined activity of various mechanisms, including decreased expression of outer membrane porins, expression of efflux pumps, and/or expression of β-lactamases, including AmpC cephalosporinases and carbapenemases (Wong et al., 2017).

Gram-negative bacteria develop resistance to β-lactams through several mechanisms (Munita et al., 2015), including the production of enzymes called β-lactamases that hydrolyse the β-lactam ring. The Ambler classification of β-lactamases, the most widely used classification system, divides β-lactamases into four classes based on their amino acid sequences (Ambler, 1980). The Ambler classes include active-site serine β-lactamases (classes A, C, and D) and metallo-β-lactamases (MBLs; class B), which require a bivalent metal ion, usually Zn^2+^, for activity (Hall and Barlow, 2005).

The production of β-lactamases, including carbapenemases, is one of the most common resistance mechanisms among Gram-negative bacteria. Most carbapenemases belong to Ambler classes A, B, or D and are chromosomal or plasmidic (Miriagou et al., 2010). Ambler class A (e.g., *K. pneumoniae* carbapenemase; KPC) is most frequently observed in Enterobacterales. Class B (MBLs, including New Delhi MBL [NDM], Verona integron-encoded MBL [VIM], imipenemase enzyme [IMP], Sao Paulo MBL [SPM], German imipenemase [GIM], Seoul imipenemase [SIM], and Dutch imipenemase [DIM]) is most frequently observed in *P. aeruginosa* and Enterobacterales. Class D, which includes oxacillinases [OXA] type-related enzymes, is most commonly observed in Enterobacterales and *A. baumannii* (Bedenić et al., 2014). To correctly manage infections caused by carbapenemase-producing microorganisms, it is necessary to know the genes encoding these enzymes.

In addition, class C includes AmpC β-lactamases, encoded by genes that can be located on plasmids or chromosome (Tamma et al., 2019; Tekele et al., 2020).

AmpC genes located on plasmids constitutively produce β-lactamases. Chromosomal AmpC produces low levels of β-lactamases but can be hyperproduced by β-lactam induction (Tamma et al., 2019). These enzymes are commonly found on Enterobacterales, *P. aeruginosa*, and *A baumannii* (Halat et al., 2016). Class C β-lactamases includes AMPC active on cephamycins [CMY-2], ApmC discovered at Dhahran [DHA], AmpC type [ACT], AmpC active on cefoxitin [FOX], Amber class C [ACC], AmpC active on moxalactam [MOX], AmpC from *Citrobacter freundii* [CFE], and *Acinetobacter*-derived cephalosporinase [ADC] (Jacoby, 2006).

### 2.1 KPC

*K. pneumoniae* is the leading producer of KPC. Strains producing KPC pose a significant threat in clinical practice (Miriagou et al., 2010; Bassetti and Peghin, 2020). It is estimated that more than 30,000 isolates of carbapenem non-susceptible Enterobacterales occur in the United States annually, and up to 81.5% of these are associated with KPC (Sader et al., 2018a; Gupta et al., 2019). Reported mortality among patients infected with KPC strains is up to 62% (Alvim et al., 2019).

### 2.2 MBLs

Metallo-β-lactamase (MBL) enzymes are class B enzymes not inhibited by clavulanic acid, sulbactam, tazobactam, or avibactam. MBLs are chromosomal and ubiquitous in some nonfermenters; however, the acquired MBLs are disseminated among the Enterobacterales and *P. aeruginosa*. The important acquired MBLs are imipenemase (IMP-1, reported in Japan), Verona integron-encoded metallo-β-lactamase (VIM, reported in Italy) and New Delhi metallo-β-lactamase (NDM, reported in India) (Boyd et al., 2020). Ambler class B β-lactamases are increasingly detected in Gram-negative bacteria, and they have a considerable clinical impact (Bush and Bradford, 2016). The incidence of MBL-producing Enterobacterales has increased in the last two decades, occurring in up to 4.3% of patients in 2009 and 12.7% in 2016 (Castanheira et al., 2004; Castanheira et al., 2019a). Rates of MBL-producing Enterobacterales vary significantly among populations; in India, MBL rates of 29%, 18.8%, and 12.3% have been reported among *P. aeruginosa*, *A. baumannii*, and Enterobacterales, respectively (Rahman et al., 2014; Rahman et al., 2018; Kloezen et al., 2021).

### 2.3 Class D, OXA Type

OXA enzymes hydrolyse carbapenems and penicillins at a low level and exhibit weak hydrolysis of second and third generation cephalosporins (Bush and Jacoby, 2010; Nordmann et al., 2011; Leonard et al., 2013). The spectrum of class D OXA β-lactamases has expanded dramatically in the last years with the addition of several new variants. This class has been detected in chromosomes and plasmids of diverse bacterial species (Pandey et al., 2021).

OXA-48-like carbapenemases are the most common carbapenemases in the Enterobacterales worldwide. The most common enzymes among the OXA-48-like carbapenemase group are OXA-48, OXA-181, OXA-232, OXA-204, OXA-162, and OXA-244, in that order.

Detection of OXA variants (such as OXA-48-like enzymes) is challenging for clinical laboratories and can be confirmed only by gene sequencing of the corresponding genes. Thus, *K. pneumoniae* and *E. coli* with OXA-48, OXA-181, and OXA-232 are of global concern and are most likely underreported due to laboratory detection problems (Pitout et al., 2019). Geographic variation of OXA-48 has been reported, with a significant presence in Turkey, the Middle East, Northern Africa, and Europe (Stewart et al., 2018; Castanheira et al., 2019a); associated with mortality levels up to 28% have been reported (Madueño et al., 2017).

Class D β-lactamases are intrinsic to many Gram-negative bacteria, including *A. baumannii*. To date, six subgroups of Class D carbapenemase enzymes have been identified in *A. baumannii*, including OXA-23, OXA-24, OXA-51, OXA-58, OXA-143, and OXA-235.

OXA-51 is natural or intrinsic among these carbapenemases, and the others are acquired (Donald et al., 2000; Poirel et al., 2010; Kamolvit et al., 2015). These enzymes are highly active against penicillins and poorly active against carbapenems. However, insertion sequence (IS) is considered a strong promoter for increasing oxacillinase expression and dissemination. It has been reported that the *ISAba1/bla*_OXA-23_ or *ISAba1/bla*_OXA-51_ combination amplifies resistance to carbapenems (Martínez and Mattar, 2012). These oxacillinases have been reported in *A. baumannii* during hospital outbreaks (Acosta et al., 2011; Tena et al., 2013).

### 2.4 AmpC β-Lactamase-Mediated Resistance

The clinical importance of AmpC β-lactamases lies in the development of resistance to penicillin, cephamycin, cephalosporin, carbapenems, and monobactams. In addition, AmpC β-lactamase activity is not altered by extended-spectrum β-lactamases (ESBL)s inhibitors. Chromosomal-mediated AmpC β-lactamases are usually found in *Serratia*, *Citrobacter*, *Enterobacter*, *Pseudomonas*, and *Acinetobacter* species, while plasmid-encoded AmpC β-lactamases are commonly found in *E. coli, K. pneumoniae, Citrobacter freundii, Salmonella* spp., and *Proteus mirabilis* (Tekele et al., 2020). AmpC β-lactamase production in Gram-negative bacilli is reported from 2.4 up to 37% in Africa, Europe and Asia (Shashwati et al., 2014; Shivana and Rao, 2017; Gómara-Lomero et al., 2018; Tekele et al., 2020). AmpC β-lactamases activity along with decreased antibiotic susceptibility by porin and efflux mechanisms expression are reported in clinical isolates (Tamma et al., 2019; Tekele et al., 2020).

## 3 New Approved Therapeutic

### 3.1 Plazomicin

Plazomicin, a new aminoglycoside antibiotic registered for use in complicated UTI, has also been used to treat CRE infection. An overall reduction in mortality (from 50% to 24%) was reported in an evaluation of plazomicin compared to colistin in combination with adjunctive meropenem or tigecycline (McKinnell et al., 2019). Further, in a large surveillance study, plazomicin was found to be active against 76.4% of MBL-producing Enterobacterales, and non-susceptibility was associated with the presence of 16S RMTase gene (aminoglycoside resistance). In most isolates, 16S-RMTase was co-expressed with NDM, resulting in heightened plazomicin resistance rates among NDM producers; specifically, 34% of NDM producers were non-susceptible to plazomicin, compared to 10.4% of non-susceptibility for VIM producers. All IMP producers were found to be susceptible to plazomicin (Serio et al., 2019). Synergistic activity evaluated *in vitro* may not always correlate with the clinical efficacy observed *in vivo*, given the different pharmacokinetic properties of the antimicrobials administered and the specific response of each individual (Iannaccone et al., 2022).

Methylases have also been reported in OXA-48 producers. In a study that included 103 pan-aminoglycoside- and carbapenem-resistant clinical isolates (35 NDM and 23 OXA-48 and 21 KPC), plasmid co-localization of the carbapenemase and the RMTase encoding genes was found among 20% of the isolates (available at https://doi.org/10.3390/microorganisms10030615).

### 3.2 Eravacycline

Eravacycline, a novel tetracycline antibiotic approved by the U.S. Food and Drug Administration (FDA) for complicated IAI, has demonstrated *in vitro* activity against CRAB (MIC_90 =_ 1 mg/l) (Seifert et al., 2018) and *in vitro* activity against CRE. However, no high-quality clinical data exist regarding the use of eravacycline to treat CRE infection (Lan et al., 2019).

### 3.3 Cefiderocol

Cefiderocol (formerly S-649266) received FDA approval for treatment of UTI in October 2019 and for treatment of hospital-acquired pneumonia and VAP in September 2020 (McCreary et al., 2021). Cefiderocol is a synthetic conjugate composed of a cephalosporin moiety and a catechol-type siderophore. The molecule binds to iron and uses active iron transporters to facilitate bacterial cell entry (Ito et al., 2018). Once inside the periplasmic space, it dissociates from iron. The structure of cefiderocol and its mechanism of cell entry may enhance protection against loss of porin channels, overexpression of efflux pumps, and inactivation by carbapenemases (Ito et al., 2018).

*In vitro* data indicates that cefiderocol has activity against MBL producers. In carbapenem-non-susceptible Gram-negative isolates, the MIC_90_ of cefiderocol was 8 mg/l for NDM producers and 4 mg/l for VIM producers (Dobias et al., 2017; Kazmierczak et al., 2019). This antibiotic has also demonstrated activity against CRPA (MIC_90_ = 0.5 mg/l) and CRAB (MIC_90_ = 1 mg/l) (Jacobs et al., 2019). In a mouse model, exposure to cefiderocol reduced bacterial 3 log10 CFU and provided a sustained kill effect at 72 h in CRAB infections (Matsumoto et al., 2017; Monogue et al., 2017; Stainton et al., 2019).

In clinical studies, cefiderocol has proven non-inferior to meropenem for the treatment of nosocomial pneumonia, with a 14-day mortality rate of 12.4% (18/145) for cefiderocol and 11.6% (17/146) for meropenem (Wunderink et al., 2021).

In another clinical trial that included 152 patients, cefiderocol was compared with the best available therapy to treat carbapenem-resistant Gram-negative bacterial infection. At all measured time points, mortality was higher in the cefiderocol group (33.7%, 34/101) than in the comparator group (18.4%, 10/49). This difference was noted in patients with pneumonia or BSI or sepsis caused by *Acinetobacter* spp. In contrast, there was no mortality difference among patients with CRE infections (Bassetti et al., 2021).

A retrospective multicenter observational study was recently performed, including patients admitted to ICU for severe COVID-19 and CRAB infections. Patients were treated with cefiderocol, as compassionate use or with alternative regimens. All-cause 28-day mortality rate was 57%, without differences between groups (*P *=* *0.70). In this study, cefiderocol was associated with a non-significant lower mortality risk (*P *=* *0.10) (Pascale et al., 2021).

Furthermore, a recent observational retrospective study that included patients with CRAB infections was performed. In this study, cefiderocol and colistin treatments were compared, and 30-day mortality was higher in patients receiving colistin than those who received cefiderocol (55.8% *versus* 34%, *P* = 0.018). This difference was confirmed in patients with BSI and not confirmed in patients with VAP (Falcone et al., 2022).

It has been proposed that multiple factors, including NDM and PER, could be related to reduced susceptibility to cefiderocol (Kohira et al., 2020). Furthermore, it has been reported that alterations in the target binding sites of *P. aeruginosa*–derived AmpC β-lactamases have the potential to reduce the activity of ceftazidime-avibactam, and cefiderocol (Simner et al., 2021)

### 3.4 Combinations of β-Lactams With β-Lactam Inhibitors

Numerous studies have indicated that combinations with new β-lactamase inhibitors **(**BLIs), such as avibactam, vaborbactam, and relebactam, may lead to superior outcomes when combined with other antibiotics (van Duin et al., 2018). The novel β-lactam-BLI combinations currently approved for clinical use have been found to be poorly active against MBL-producing strains.

#### 3.4.1 Ceftazidime-Avibactam

Avibactam is a non-β-lactam BLI inhibitor of class A, C, and some class D β-lactamases. It is combined with ceftazidime to restore its activity against isolates of carbapenem-resistant Gram-negative bacilli carrying KPC and OXA-48 (Soriano et al., 2021). However, it is not active against MBL-producing strains (Shirley, 2018). In the EU, ceftazidime-avibactam is approved to treat adults with complicated UTIs, including pyelonephritis, VAP, and other infections caused by Gram-negative species. This combination has a tolerability and safety profile similar to ceftazidime alone (Pharmacy Benefits Management Services, 2015). *In vitro* evidence indicates that ceftazidime-avibactam is active against OXA-48-producing Enterobacterales (n = 265, MIC_90_ = 4 mg/L, 92.5% susceptibility) (García-Castillo et al., 2018; Kazmierczak et al., 2018). Further, *in vitro* activity of ceftazidime-avibactam against CRE collected from urine specimens (n = 11,826) was evaluated, and this combination demonstrated 100% susceptibility in KPC and OXA-48 producers (Pitout et al., 2019).

Clinical evidence indicates that ceftazidime-avibactam is superior to colistin-based regimens for the treatment of CRE-infected patients (46% of them with BSI) (van Duin et al., 2018). The former combination has been associated with lower in-hospital 30-day mortality than the latter (Shields et al., 2017; Tumbarello et al., 2019). Additionally, the efficacy of ceftazidime-avibactam was evaluated in a study of 24 patients with several OXA-48 CPE infections. The results indicated a 30-day mortality rate of 8.3% and a clinical cure rate of 62.5%. Seven of the 24 patients (35%) had infection recurrence at 90 days (De la Calle et al., 2019). Ceftazidime-avibactam has been successfully used in salvage therapy to increase clinical cure occurrence, increase microbiological cure occurrence, and increase the patient likelihood of survival to discharge from the hospital (Stewart et al., 2018).

Ceftazidime-avibactam has been combined with other antibiotics; for example, the addition of fosfomycin to ceftazidime-avibactam has been found to reduce synergy significantly and bacterial burden compared to the use of ceftazidime-avibactam alone in a murine infection model of infection by MDR *P. aeruginosa* (Livermore et al., 2017; Papp-Wallace et al., 2019). Other antibiotics—including amikacin, colistin, meropenem, and aztreonam—have also demonstrated synergy against MDR *P. aeruginosa* when added to ceftazidime-avibactam (Mikhail et al., 2019).

Ceftazidime-avibactam is a valuable treatment option for difficult-to-treat infections and is significantly associated with KPC carbapenem-resistant strains. However, MBL-producing organisms are resistant to ceftazidime-avibactam. The widespread use of ceftazidime-avibactam has been associated with a change in the epidemiology of carbapenemases from KPC to MBL (Papadimitriou-Olivgeris et al., 2019). Resistance rates reported to ceftazidime-avibactam for Enterobacterales are <2.6% (Wise et al., 2018, Wang et al., 2020, Cavallini et al., 2021) and up to 8% for *P. aeruginosa* (Nichols et al., 2016).

#### 3.4.2 Ceftolozane-Tazobactam

Tazobactam is a potent BLI of most common class A and C β-lactamases. This BLI binds to chromosomally and plasmid-mediated bacterial β-lactamases (Snydman et al., 2014).

In the existing clinical evidence, ceftolozane-tazobactam has demonstrated good activity against CRPA (Shortridge et al., 2018). Treatment with ceftolozane-tazobactam has demonstrated higher clinical cure rates than polymyxin or aminoglycoside-based regimens in the treatment of MDR-*P. aeruginosa* infections (Pogue et al., 2020). In the ASPECT-NP trial, mechanically ventilated participants with VAP were randomized to receive 3 g of ceftolozane-tazobactam (2 g), ceftolozane-tazobactam (1 g), or 1 g meropenem. This study included 511 patients, 264 of whom were treated with ceftolozane-tazobactam and 247 with meropenem. Causative agents included *K. pneumoniae* (34.6%), *P. aeruginosa* (25.0%), and *E. coli* (18.2%). The all-cause mortality at 28 days was 20.1% and 25.8%, respectively, and clinical cure rates were 60.6% and 57.1%, respectively. Microbiological eradication of Enterobacterales (74.4% and 69.7%, respectively) and *P. aeruginosa* (74.6% and 63.1%, respectively) were comparable (Martin-Loeches et al., 2022).

#### 3.4.3 Imipenem-Relebactam

Relebactam is a novel diazabicyclooctane BLI that inhibits Ambler class A β-lactamases, including TEM-type, SHV-type and CTX-M-type ESBLs, KPCs, and class C β-lactamases (Livermore et al., 2013; Livermore et al., 2018).

Imipenem-cilastatin/relebactam, a β-lactam-BLI, was approved in 2019 to treat adults with VAP, complicated UTIs, including pyelonephritis, or complicated IAI caused by susceptible Gram-negative bacilli (https://www.accessdata.fda.gov/drugsatfda_docs/label/2020/212819s002lbl.pdf).

Imipenem-relebactam has shown good activity against carbapenem-resistant isolates, including KPC producers. The use of imipenem-relebactam compared to colistin with imipenem in infections by strains that are not susceptible to imipenem showed an improved clinical response and mortality rate (Motsch et al., 2020).

A randomized controlled, double-blind Phase 3 trial evaluated the efficacy and safety of imipenem-cilastatin-relebactam to treat hospital-acquired VAP. The results indicate that this combination is not inferior to piperacillin-tazobactam (< 0.001). The 28-day all-cause mortality rate was 15.9% with imipenem-cilastatin-relebactam and 21.3% with piperacillin-tazobactam (Titov et al., 2021). Another randomized (1:1:1) controlled Phase 2 trial compared the administration of imipenem-cilastatin-relebactam (250 mg), imipenem-cilastatin-relebactam (125 mg), and imipenem-cilastatin alone in adults with complicated UTI or acute pyelonephritis and found clinical cure rates of 97.1%, 98.7%, and 98.8%, respectively (Sims et al., 2017; Motsch et al., 2020).

The epidemiology of BSI by carbapenemase-producing *K. pneumoniae* among ICU patients indicates that MBLs were more frequent in 2018 than in 2015–17 (51% and 12%, respectively; *p *< 0.001). Prior administration of ceftazidime/avibactam was independently associated with BSI development due to ceftazidime/avibactam-resistant isolates (*p* = 0.014) (Papadimitriou-Olivgeris et al., 2019).

#### 3.4.4 Meropenem-Vaborbactam

Vaborbactam is a cyclic boronic acid derivative approved by the FDA in 2017 to be used in combination with meropenem. It is a non-suicidal inhibitor and lacks antibacterial activity (Hecker et al., 2015, Lomovskaya et al., 2017). A Phase 3 open-label randomized controlled trial conducted from 2014 to 2017 (TANGO II) evaluated the efficacy and safety of meropenem-vaborbactam monotherapy compared to the best available therapy for treating CRE. This study included 77 patients with confirmed or suspected CRE infection (i.e., bacteremia, VAP, complicated UTI, or acute pyelonephritis). Cure rates were 65.6% (21/32) and 33.3% (5/15). All-cause mortality at day 28 was 15.6% (5/32) for meropenem-vaborbactam and 33.3% (5/15) for the best available therapy. Compared with the best available therapy, meropenem-vaborbactam therapy for CRE infection was associated with an increased clinical cure rate, decreased mortality, and reduced nephrotoxicity (Wunderink et al., 2018).

## 4 New Treatments in Development

### 4.1 β-Lactam and β-Lactam Inhibitors

#### 4.1.1 Cefepime-Zidebactam

Cefepime is a broad-spectrum β-lactam antibiotic studied *in vitro* for administration with BLIs, including zidebactam (cefepime-zidebactam, 8/8 mg/L). Zidebactam is a non-β-lactam agent with two mechanisms of action involving selective and high-affinity Gram-negative PBP2 binding and β-lactamase inhibition (Sader et al., 2017), with reported activity against Enterobacterales and *P. aeruginosa*-producing KPCs, AmpC, and MBLs (Sader et al., 2017). *In vitro* evidence has indicated that Enterobacterales are susceptible to cefepime-zidebactam (MIC_50/90_ = 0.25/1 mg/L for strains harboring KPC, MIC_50/90_ = 0.5/8 mg/L for strains with VIM, IMP, and NDM encoding genes).

This combination is also active against *P. aeruginosa* (MIC_50/90_ = 4/8 mg/L for strains encoding VIM and IMP genes) (Jean et al., 2022). Another study found that cefepime-zidebactam inhibits most MBL-positive Enterobacterales (90.5%, 190/210) and MBL-positive *P. aeruginosa* (94.5%, 97/103) (Mushtaq et al., 2021). Further, this combination showed moderate activity against OXA-23/24/58-producing *A. baumannii* (MIC_50/90_ = 32/32 mg/L) (Sader et al., 2017; Mushtaq et al., 2021).

#### 4.1.2 Cefepime-Taniborbactam

Taniborbactam (VNRX-5133) is a recently developed BLI with a broad spectrum of activity. The molecule is a cyclic boronate BLI that, when combined with cefepime, has activity against β-lactamase-producing CRE and CRPA and has direct inhibitory activity against Ambler class A, B, C, and D enzymes (Hamrick et al., 2020). *In vitro* studies indicate that cefepime-taniborbactam is a potential therapeutic option for patients infected with CRE (n = 247, 97.6%) and CRPA isolates (n = 170, 67.1%). This combination has demonstrated activity against Enterobacterales isolates with serine-β-lactamases of Classes A and D (97.2%) and MBL producers (93.5%) (Hernández-García et al., 2022a). Furthermore, cefepime-taniborbactam demonstrated 88.6% activity against strains of meropenem-resistant *Pseudomonas* spp. with genes encoding serine-β-lactamases (Sader et al., 2017; Hernández-García et al., 2022b).

Two clinical studies are ongoing, and results are expected to be published: A Phase 3 randomized, double-blind, active-controlled non-inferiority clinical study that evaluated the efficacy, safety, and tolerability of cefepime-taniborbactam in adults with complicated UTI, including acute pyelonephritis (available at https://clinicaltrials.gov/ct2/show/study/nct03840148) and a Phase 1 open-label single-dose study that evaluated the pharmacokinetics, safety, and tolerability of taniborbactam with VNRX-5022 in subjects, including 33 participants with renal impairment (available at https://clinicaltrials.gov/ct2/show/nct03690362).

#### 4.1.3 Aztreonam-Avibactam

Aztreonam is stable to hydrolysis by MBLs; however, aztreonam can be inactivated by ESBLs, KPCs, and AmpC β-lactamases. Thus, Gram-negative bacilli harboring MBL that carry other β-lactamases can inactivate aztreonam (Karlowsky et al., 2017a). The combination of aztreonam-avibactam is currently under clinical development for the treatment of serious infections caused by class A and C β-lactamases–producing Enterobacterales (Mauri et al., 2021). In this combination, aztreonam is the active agent and avibactam act as BLI, which protects aztreonam against these β-lactamase types (Mauri et al., 2021).

Aztreonam-avibactam has shown potent *in vitro* activity against MBL-positive Enterobacterales isolates, for which there are few treatment options. An analysis of 267 Enterobacterales isolates collected from 2012–2015 that harbored MBL genes (NDM, VIM, and IMP) found an MIC_90_ = 1 mg/L for aztreonam-avibactam. Regarding MBL-positive *P. aeruginosa* isolates (n = 452), the MIC_90_ for aztreonam-avibactam was 32 mg/L (Karlowsky et al., 2017b).

A Phase 2a open-label multicentre clinical study found that the use of aztreonam-avibactam with metronidazole to treat adults with complicated IAI was well tolerated (Cornely et al., 2020). Recently, aztreonam avibactam resistance was reported in 15 of 110 carbapenem-resistant NDM and OXA producing *E. coli* (Nordmann et al., 2021)

#### 4.1.4 Aztreonam and Ceftazidime-Avibactam

*In vitro* data support the claim that a combination of aztreonam and ceftazidime-avibactam is an effective treatment for infections caused by MBL producers; however, clinical studies are lacking. Aztreonam susceptibility in combination with ceftazidime-avibactam was evaluated in 50 MBL-producing Enterobacterales using the Etest strip superposition method. In these assays, aztreonam susceptibility was restored in 86% of the MBL-producing Enterobacterales isolates when combined with ceftazidime-avibactam (Emeraud et al., 2019).

Another study evaluated this combination against 21 carbapenem-resistant *K. pneumoniae* strains and 21 MDR *P. aeruginosa* strains. Compared to ceftazidime-avibactam alone, a fourfold decrease in ceftazidime-avibactam MICs was observed for most *K. pneumoniae* strains, and a twofold or greater reduction for most *P. aeruginosa* isolates in most of the combinations evaluated. In both *P. aeruginosa* and *K. pneumoniae* strains, the combination of ceftazidime-avibactam with aztreonam was synergistic (≥ 2.15-log_10_ CFU/ml decrease) (Mikhail et al., 2019).

Clinical evidence indicates that the aztreonam and ceftazidime-avibactam combination provides a therapeutic advantage compared with other active antibiotics for patients with BSI due to MBL-producing Enterobacterales. In a study of 102 patients with BSI (79 NDM-producing and 14 VIM-producing *K. pneumoniae*, 3 NDM-producing *E. coli*, 5 VIM-producing *Enterobacter* spp., 1 VIM-producing *Morganella morganii*), the use of aztreonam and ceftazidime-avibactam was associated with lowered 30-day mortality (p = 0.01), shortened length of stay (p = 0.007), and lowered clinical failure at day 14 (p = 0.002) (Falcone et al., 2021). Further, the aztreonam and the ceftazidime-avibactam combination has been used in clinical practice to successfully treat infections caused by MBL-producing Enterobacterales based on their *in vitro* synergy (Marshall et al., 2017; Avery and Nicolau, 2018; Jayol et al., 2018).

Aztreonam has been paired with several other β-lactam-BLI combinations to determine its efficacy against MBL producers. For example, the ticarcillin-clavulanate–aztreonam combination was found to be synergistic or additive against SPM-1-positive *P. aeruginosa* (Rocha-Santos et al., 2021), whereas combinations with ceftolozane-tazobactam did not demonstrate the same effect (Cuba et al., 2020).

In another study, combinations of aztreonam with ceftazidime-avibactam, ceftolozane-tazobactam, and amoxicillin-clavulanate were evaluated against MBL-positive Enterobacterales and *P. aeruginosa*. Ceftazidime-avibactam proved to be the most potent combination, restoring aztreonam susceptibility in 86% of the Enterobacterales isolates. Amoxicillin-clavulanate restored aztreonam activity in 50% of isolates and ceftolozane-tazobactam in 20% of isolates (Emeraud et al., 2019).

In addition, patients with complicated UTIs caused by NDM-producing *E. coli* and respiratory tract infection associated with XDR *S. maltophilia* have been successfully treated with aztreonam–ceftazidime-avibactam and aztreonam–amoxicillin-clavulanate combinations, respectively (Emeraud et al., 2019). However, additional clinical studies are needed to determine the optimal combination regimen for this promising therapeutic approach, including timing and dosage.

#### 4.1.5 QPX7728 With Meropenem and Other Antibiotics

QPX7728 is a novel ultrabroad-spectrum cyclic boronic acid BLI that has demonstrated activity against serine and MBLs when used in combination with β-lactam antibiotics. The activity of QPX7728 in combination with multiple β-lactams against carbapenem-resistant *K. pneumoniae* isolates was evaluated in a neutropenic mouse infection model. The combination of QPX7728 and aztreonam, biapenem, cefepime, ceftazidime, ceftolozane, and meropenem produced bacterial killing at all QPX7728 doses evaluated. These data indicate that QPX7728 administered in combination with different β−lactam antibiotics may have utility in treating bacterial infections due to carbapenem-resistant *K. pneumoniae* (Sabet et al., 2020). Further, QPX7728, in combination with cephalosporins or carbapenems, including meropenem, was found to have excellent activity against CRE-producing serine or MBL (Nelson et al., 2020; Lomovskaya et al., 2021).

#### 4.1.6 Durlobactam-Sulbactam

Durlobactam (formerly ETX2514) is a new member of the diazabicyclooctane class of β-lactamase inhibitors with broad-spectrum activity against Ambler class A, C, and D serine β-lactamases. Sulbactam (a semi-synthetic penicillanic acid) is a β-lactamase inhibitor with limited activity to class A serine β-lactamases; however, it possesses intrinsic antibacterial activity against *Acinetobacter* (Shapiro et al., 2021). Durlobactam-sulbactam- is a combination that has demonstrated potential to treat CRAB effectively (Durand-Réville et al., 2017).

*In vitro* activity of durlobactam-sulbactam on MDR *A. baumannii* isolates has been reported, with MIC90 values ranging from 2-4 to 4-4 mg/L. The *in vivo* efficacy has been demonstrated in murine infection models against XDR *A. baumannii* with MIC values from 0.5-4 to 4-4 mg/L (Shapiro et al., 2021).

Based on observed *in vitro* activity, three Phase 1 trials, one Phase 2 trials, and one Phase 3 study (the ATTACK trial) are ongoing (available at: https://clinicaltrials.gov/ct2/show/NCT03894046).

#### 4.1.7 Nacubactam

Nacubactam is a BLI that belongs to the new class of diazabicyclooctane. It inhibits *E. coli* penicillin-binding protein 2 (PBP2) (Barnes et al., 2019) and acts synergistically as a β-lactam enhancer when combined with β-lactams. This enhancer effect results from these combinations possessing the ability to target multiple PBPs (Barnes et al., 2019).

*In vitro* antimicrobial activity against several classes of β-lactamase-producing Enterobacterales has been reported. Has been combined with β-lactams (aztreonam, cefepime, and meropenem) against four *Enterobacter cloacae* and 6 K*. pneumoniae* isolates in a murine model of pneumonia. Combination therapies showed enhanced antimicrobial activity (compared with monotherapies) against carbapenem-resistant *E. cloacae* and *K. pneumoniae* (strains carried IMP-1, IMP-6, or KPC genes) (Hagihara et al., 2021).

#### 4.1.8 ETX1317-Cefpodoxime

ETX1317 is a novel diazabicyclooctane that has been found to restore the antibacterial activity of several classes of β-lactams, including third-generation cephalosporins, with broad inhibition of classes A, C, and D serine β-lactamases, including CRE (O'Donnell et al., 2020). ETX1317 is an oral prodrug (ETX0282) that is administered with cefpodoxime proxetil, and it has demonstrated oral efficacy in murine models of infection. The available evidence indicates that orally administered combinations may be helpful in treating CRE (Miller et al., 2020).

### 4.2 Polymyxins

#### 4.2.1 SPR741

SPR741 (formerly NAB741) is a polymyxin-B-derived molecule intended to minimize the nephrotoxicity associated with this antibacterial class by reducing its positive charge (Impey et al., 2020). *In vitro* evidence indicates that SPR741-rifampin has a MIC of 128 mg/L-4.0 mg/L against *A. baumannii*. Synergistic activity was detected using the checkerboard method. The MIC of rifampin decreased from 4.0 to 0.5 mg/L in the presence of 2.0 mg/L SPR741 (Zurawski et al., 2017).

In combination therapy with azithromycin, SPR741 showed promising *in vivo* activity against MDR *Enterobacteriaceae* isolates with an azithromycin MIC of ≤ 16 mg/l These data support a potential role for azithromycin-SPR741 in the treatment of MDR *Enterobacteriaceae* infection (Stainton et al., 2018).

The combination of rifampin and SPR741 has proven to be active against MBL producers. However, the rapid development of resistance to rifampin may hinder the further development of this combination (Vaara, 2019). Further, several b-lactam-SPR741 combinations have been evaluated against Enterobacterales isolates, including MBL producers, and mecillinam-SPR741 demonstrated the most significant activity (Mendes et al., 2020). A Phase 1 clinical trial of SPR741 indicated that the drug was well tolerated in healthy volunteers (Eckburg et al., 2019).

#### 4.2.2 SPR206

SPR206 is a novel polymyxin derivative that has demonstrated potent *in vitro* and *in vivo* activity against *A. baumannii*, *P. aeruginosa*, and multiple clinically important species of *Enterobacterales*, including MDR ESBL-producing and β-lactamase-producing strains (from Ambler classes A, B, C, and D). *In vitro* studies have found that SPR206 exhibits lower MIC (0.12–0.5mg/ml) than colistin and meropenem against *A. baumannii, Klebsiella pneumoniae, and P. aeruginosa* (Zhang et al., 2020).

Non-clinical toxicology studies in mice, rats and non-human primates have found that SPR206 has a lower risk for kidney toxicity than colistin and polymyxin B (Brown et al., 2019; Zhang et al., 2020). A double-blind placebo-controlled single and multiple-ascending-dose study of the safety, tolerability, and pharmacokinetics of SPR206 was conducted on 94 healthy subjects, and SPR206 was generally safe and well-tolerated (Bruss et al., 2021).

#### 4.2.3 QPX9003

QPX9003 is a novel polymyxin analogue with an improved safety profile compared to current polymyxins. QPX9003 was evaluated against *P. aeruginosa* (n = 1,000), CRAB (n = 503), and Enterobacterales (n = 1,105). The molecule showed activity against *P. aeruginosa* and was four times more active than colistin (MIC_50/90_ = 0.25/0.25 for QPX9003 and MIC_50/90_ = 1/1 for colistin). QPX9003 also showed higher activity than colistin against CRAB (MIC_50/90_ = 0.125/1 for QPX9003 and MIC_50/90_ = 0.5/4 for colistin). Additionally, QPX9003 showed a modal MIC of 0.06 mg/L against Enterobacterales isolates resistant to cephalosporins and/or carbapenems (MIC_50/90_ = 0.06/16) (Castanheira et al., 2019b). The observed activity of QPX9003 and colistin were similar to those of Enterobacterales (Castanheira et al., 2019b). A Phase 1 randomized double-blind placebo-controlled ascending single and multiple-dose clinical study of the safety, tolerability, and pharmacokinetics of intravenous QPX9003 in 80 healthy adult subjects is ongoing (available at https://clinicaltrials.gov/ct2/show/NCT04808414).

### 4.3 Other Membrane Protein Targeting Antibiotics

#### 4.3.1 Murepavadin (POL7080)

Murepavadin (POL7080) is a member of a novel class of outer-membrane-protein-targeting antibiotics developed to treat resistant *P. aeruginosa* infection. In an evaluation of 300 MDR and 167 XDR-*P. aeruginosa* isolates, murepavadin had MIC_50/90_ = 0.12/0.25 mg/l for both groups (Sader et al., 2018b). Additionally, potent activity was exhibited by murepavadin in 785 infections due to XDR *P. aeruginosa* isolates, with MIC ≤ 0.5 mg/l in 96.7% of isolates (Sader et al., 2018c). However, Phase 3 trials to evaluate this antimicrobial in the treatment of nosocomial (PRISM-UDR) and VAP (PRISM-MDR) have been suspended because safety data revealed unacceptably high rates of kidney injury in the murepavadin group (available at: https://clinicaltrials.gov/ct2/show/NCT03409679).

## 5 Concluding Remarks

New antimicrobial drugs and combinations have recently been approved to treat carbapenem-resistant Gram-negatives, and some others are in development. These new alternatives should be carefully used to maintain their usefulness and minimize resistance generation.

The treatment of infections by organisms encoding MBLs remains the most problematic because few drugs are helpful for these strains. It is necessary to continue the research of new antimicrobials to treat carbapenem-resistant gram negatives.

## Author Contributions

Conceptualization, data analysis, and writing of draft manuscript by EG-G. Review and editing manuscript by AM-M, RM-O, ER-N, HJM-G, and FC-L. All the authors defined research topic developed for this manuscript have read and agree to the final version.

## Conflict of Interest

The authors declare that the research was conducted in the absence of any commercial or financial relationships that could be construed as a potential conflict of interest.

## Publisher’s Note

All claims expressed in this article are solely those of the authors and do not necessarily represent those of their affiliated organizations, or those of the publisher, the editors and the reviewers. Any product that may be evaluated in this article, or claim that may be made by its manufacturer, is not guaranteed or endorsed by the publisher.
